# Real-Time Biophysical Phenotyping and Sorting of Transiting Cells Along a Constriction Microchannel

**DOI:** 10.3390/mi17080936

**Published:** 2026-08-06

**Authors:** Zhongning Jiang, Wei Huang, Jingqian Zhang, Raymond H. W. Lam

**Affiliations:** 1 Department of Biomedical Engineering, College of Biomedicine, City University of Hong Kong, Hong Kong 999077, China; zhonjiang8-c@my.cityu.edu.hk (Z.J.); whuang42-c@my.cityu.edu.hk (W.H.); jzhang3325-c@my.cityu.edu.hk (J.Z.); 2 China Resources Research Institute of Life Sciences and Health, Shenzhen 518045, China; 3 City University of Hong Kong Shenzhen Research Institute, Shenzhen 518057, China

**Keywords:** cell, sorting, phenotyping, channel, microfluidic

## Abstract

We present a microfluidic platform for real-time, label-free cytometry and sorting of single cells based on intrinsic biophysical properties. The system integrates impedance-based electrokinetic sensing with a constriction microchannel architecture to induce controlled deformation during cell transit. Electrical signals captured via lock-in amplification are processed through a parallel software pipeline incorporating deep learning algorithms for event detection and feature extraction. A biomechanical model enables conversion of raw signal features into cell size and whole-cell elasticity, validated against imaging measurements. Experimental results demonstrate accurate phenotyping and sorting of live and dead MCF-7 cells, achieving sorting accuracy of 84%. This approach offers a cost-effective and scalable solution for biophysical analysis, with potential applications in liquid biopsy, disease diagnostics, and personalized medicine.

## 1. Introduction

Biophysical properties of cells provide valuable insights into their physiological and pathological states [1]. These properties, when correlated with disease conditions, serve as important biomarkers for diagnosis and research [2]. Recent advances have enabled automated physical phenotyping and sorting at the single-cell level, offering new opportunities for label-free cellular analysis.

Among existing techniques, deformability cytometry is widely used to assess cell deformation under hydrodynamic stress through imaging [3,4,5,6]. Other approaches measure cell transit speed through microchannel constrictions [7,8], using optical imaging [9] or electrokinetic sensing [10]. These methods have demonstrated strong classification performance, achieving up to 95% accuracy in distinguishing malignant from benign cells [11]. Furthermore, electrical signal features have been shown to correlate with cellular mechanical properties [12].

Electrokinetic sensing, in principle, offers fast measurements suitable for liquid biopsy applications [13,14]. Compared to laser-based techniques, which are costly due to expensive optical components, and image-based methods, which are limited by camera frame rates and resolution, electrokinetic sensing provides a more scalable solution. Typically, this approach involves detecting signal peaks generated as cells pass over electrodes and converting these events into biophysical parameters [15]. However, signal integrity can be affected by factors such as cell surface protein composition, highlighting the need for robust and efficient measurement schemes. Some platforms have attempted to decouple interdependent properties. For example, Petchakup et al. employed impedance-based deformability cytometry to characterize neutrophils using normalized electrical metrics [12], while Liang et al. combined flow-stretching imaging with impedance sensing and machine learning, achieving classification accuracies between 83.1% and 95.4% [16,17]. Despite these advances, many methods require prior knowledge of cell geometry and flow conditions, limiting their applicability to unknown or heterogeneous cell populations.

Signal processing algorithms have also evolved to improve event detection. Caselli et al. [18] proposed a model-based approach that fits signal shapes to extract features, but its performance degrades under unstable conditions such as channel blockage or pressure fluctuations. It has been reported that deep learning methods can offer more robust measurement, with outstanding performance even under a low signal-to-noise ratio condition [19]. Deep learning methods [20], including long short-term memory-based networks [21], have been applied to capture events from time series data, yet uneven cell distribution in suspension often leads to high false-positive rates. Additionally, many reported systems employ serial software architectures [15], introduce delays and hinder real-time processing performance.

Microfluidic sorting technologies have also progressed significantly [22]. For instance, Hu et al. [23] integrated immunofluorescence staining with dielectrophoresis for marker-specific sorting of rare cells. Acoustofluidic techniques have been applied for sorting cells. Acoustofluidic techniques have been applied for sorting of cells [24], droplets [25] and particles [26]. Pneumatic microvalves achieved with bilayer channels [27] have shown improved sorting performance and preserved cell viability [28]. It can be explained by that, unlike electrokinetic and acoustic forces, this method generates shear stress over the cell surface but does not cause intracellular stimulations.

In this work, we present a microfluidic platform that integrates real-time cell elasticity cytometry with active sorting. Recently, we have reported a microfluidic cell elasticity cytometry [29] using electrokinetic sensing, yet a feasible cell sorting scheme has not yet been developed for the extended cell screening application of the microfluidic platform. Therefore, we attempt to implement cell sorting by integrating microfluidic pneumatic valves with minimal intracellular stimulations, followed by examining the resultant sorting performance. The system comprises two main components: (1) a microfluidic device for impedance-based sensing and flow path control and (2) software architecture for real-time signal analysis and cell classification. Electrical signals are captured as cells pass through electrodes embedded along constriction microchannels. Events are detected using a deep learning algorithm, and cell properties are computed based on a theoretical model. Classification is performed using a linear decision boundary for rapid processing, and pneumatic microvalves are actuated to direct cells to designated outlets. This approach enables automated, label-free sorting based on intrinsic biophysical properties.

## 2. Materials and Methods

### 2.1. Device Fabrication

The microfluidic device contains two layers of microchannels with electrodes patterned over the base of the lower channels (flow channels). The fabrication is based on replica molding of polydimethylsiloxane (PDMS) and photolithography for the electrodes. The required mold was produced by patterning a layer (thickness: 40 μm) of negative photoresist (SU-8 2015, Kayaku Advanced Materials, Worcester County, MA, USA) on a silicon wafer with the device design using standard photolithography procedures. Next, a PDMS prepolymer was prepared by mixing the base and the curing agent with a weight ratio of 10:1, followed by degassing in a vacuum chamber. The prepolymer was spin-coated on the mold for the flow channels (1000 rpm for 60 s), while the prepolymer was poured on the mold for the upper channels (gas channels) for a thickness of ~4 mm, contained in a Petri dish.

Then, both molds with PDMS were cured in an oven at 85 °C overnight. After cutting and peeling off the gas channels substrate, its patterned surface was treated with air plasma (energy: 5 kJ; PDC-002, Harrick Plasma, Ithaca, NY, USA) and aligned onto the flow channel substrate under a dissecting microscope. The bound substrate was then cut and peeled off, followed by punching holes via it for the device inlets and outlets.

On the other hand, the required electrodes were fabricated by patterning a 1 μm thick photoresist (AZ5214, AZ electronics, Concord, ON, USA) on a gold-coated (thickness: 100 nm) glass slide (Fuzhou Innovation Photoelectric Technology Co., Ltd., Fuzhou, China) using the standard photolithography procedures. Photoresist AZ5214 with thickness 1.2 μm is spin-coated on the glass, followed by a bake step at 110 °C for 1 min. Subsequently, the photoresist is covered by a mask with pattern of electrodes and exposed to ultraviolet light with energy density of 120 mJ/cm^2^. After developing the photoresist in an AZ400k mixed solution with water ratio of 1:3 for about 30 s, the aluminum layer is covered with the protective electrode pattern photoresist. After removing the exposed gold regions with the corresponding etchant (KI:I_2_:H_2_O at a concentration of 4 g:1 g:40 mL) and the underneath titanium adhesion layer with 70% oxalic acid) for 30 min at 75 °C, the AZ photoresist was also striped by acetone. Afterward, the double layer PDMS substrate was bonded to an electrode substrate using air plasma (energy: 5 kJ; Harrick plasma cleaner PDC-002) with alignment under a dissecting microscope.

To render the measuring region in the microfluidic chip inert, non-adhesive, block copolymer pluronics F-127 (Sigma-Aldrich, St. Louis, MO, USA) was injected into the device for 30 min to allow coating of pluronics F-127 on PDMS walls.

### 2.2. Electrical Signal Acquisition

The reported electrical sensing scheme relies on the impedance sensing between electrodes fabricated along microchannels in a microfluidic device. A set of electrodes in a measurement region of the device was connected to a lock-in amplifier (MFLI, Zurich instruments, Zurich, Switzerland). The center electrode was connected to the excitation signal port; and the upstream and downstream electrodes were connected to the two voltage differential input ports of the lock-in amplification machine. The excitation signal had an oscillating frequency (in the range of 10^2^ kHz) and a peak–peak voltage (in the range of 1 mV). A low-pass filter with a cutoff frequency of 20 kHz was also set with the lock-in amplification. Signal acquisition was achieved with a Python application programming interface (API) (sampling rate: ~50 kHz). We wrote Python (version 3.13) scripts, with artificial intelligence models, to extract *V_pp_* and *t_pp_* for the measurement regions and to implement the real-time conversion of cell properties.

### 2.3. Cell Preparation

Human breast cancer adenocarcinoma cells (MCF-7) are all obtained from American Type Culture Collection (Manassas, VA, USA). The cells were cultured in a high-glucose Dulbecco’s modified Eagle’s medium (Thermal Fisher Scientific, Waltham, MA, USA). They were maintained at 37 °C with saturated humidity and 5% carbon dioxide. Upon ~80% confluency of the cell population, trypsin-ethylenediaminetetraacetic acid (0.25%) was applied to resuspend cells for subculture (cell-seeding density: 5 × 10^4^ cells/cm^2^). We prepare the ‘dead’ cells by incubating them at 60 °C in a water bath for 1 h.

For cell viability test, the cells are stained by the LIVE/DEAD™ cell imaging kit (cat# R37601, Thermal fisher; 1/1000 dilution from the stock staining solutions) for 15 min at 25 °C, inducing fluorescence for live (GFP) and dead (Cy3) cells.

### 2.4. Microscopic Imaging

For microstructures in the scale of ~100 µm, we utilized an inverted fluorescence microscope (TE300, Nikon, Tokyo, Japan) installed with a scientific complementary metal oxide semiconductor camera (Zyla 4.2, Andor, Belfast, UK) for microscopic imaging and a customized environment chamber for maintaining temperature (37 °C), humidity (saturated), and gas (5% carbon dioxide) conditions.

## 3. Model

**Figure 1:**
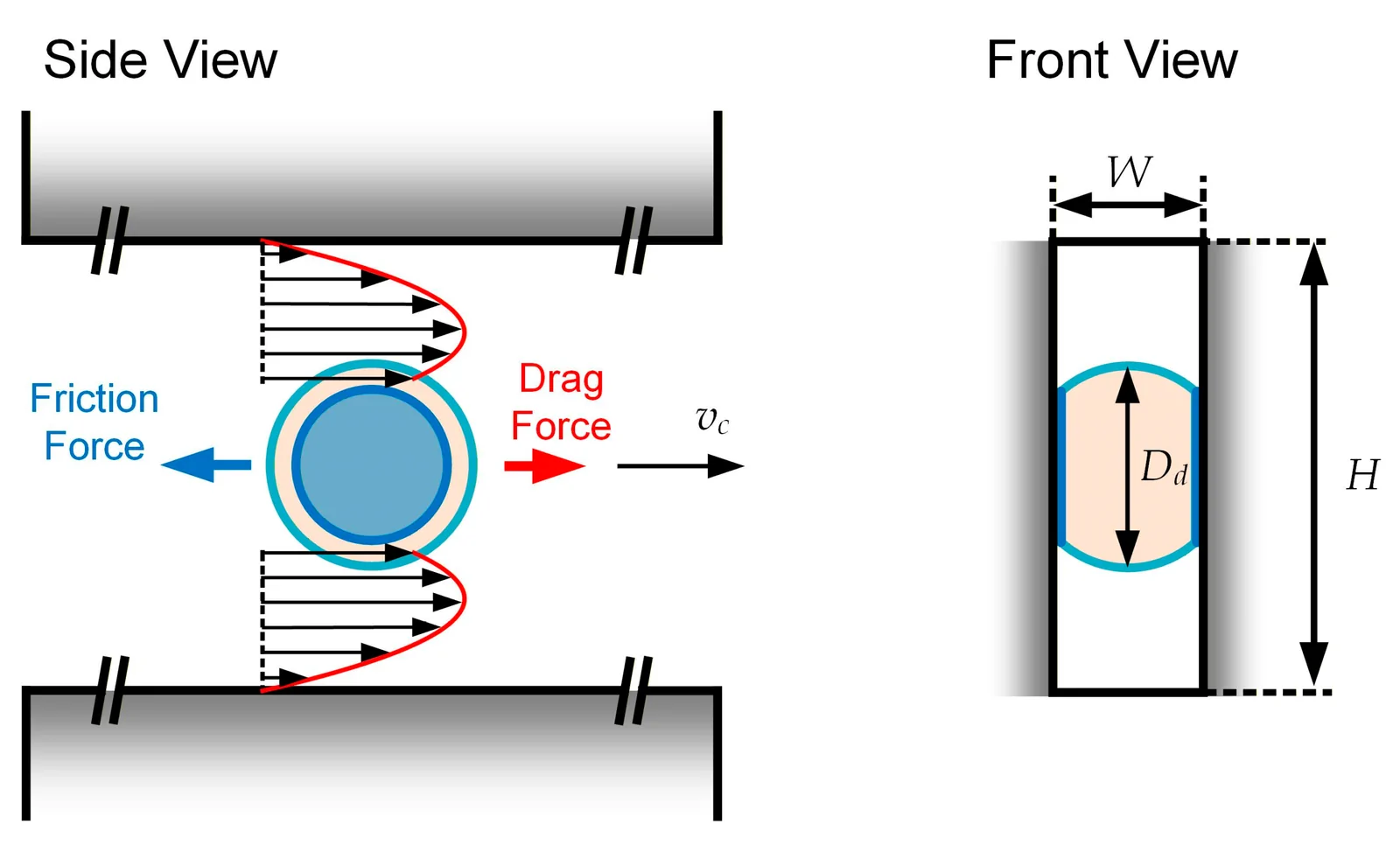
Key parameters of a deformed cell transiting along a constriction microchannel. A constant transit speed (*v_c_*) is achieved with balance of the drag force (red arrow) and the friction force (blue arrow). The drag force is caused by the surrounding flow; and the friction force (blue arrow) from the contact area between the deformed cell (with a deformed diameter denoted as *D_d_*) and the channel sidewalls. The channel height and width are denoted as *H* and *W*, respectively.

To quantify the relationship between cell transit speed and its intrinsic properties, we developed a theoretical model describing the motion of a cell through a constricted microchannel. The channel geometry is defined by width *W* (along the *y*-axis) and height *H* (along the *z*-axis), as illustrated in Figure 1. A deformed cell of initial diameter *D_c_* is approximated as a truncated sphere compressed by the channel walls [30]. The effective deformed diameter *D_d_* depends on the undeformed cell size and the constriction width, expressed as:

(1) $$D_{d}=\sqrt{\frac{2{D_{c}}^{3}}{3W}+\frac{W^{2}}{3}}$$

Recently, we have derived an expression of the transit speed (*v_c_*) based on the balance of drag and friction forces acting on a transiting cell [29]. In this work, cell deformation is approximated using the Hertz model [31] for the more straightforward computation. *v_c_* can be expressed as:

(2) $$v_{c}\sim \frac{\gamma }{4\eta }{\left[\left(\zeta H-\frac{\chi \pi D_{d}}{4+{2\left(W/D_{c}\right)}^{3}}\right)\right]}^{2}-\frac{\mu E\sqrt{D_{c}{\left(D_{c}-W\right)}^{3}}}{3\left(1-\upsilon^{2}\right)\eta WD_{d}}\left(\zeta H-\frac{\chi \pi D_{d}}{4+2{\left(W/D_{c}\right)}^{3}}\right)$$

where *γ* represents the average pressure drop per unit length along constriction channels, *η* is the liquid viscosity (~10^−3^ Pa·s for water), *H* is channel height, *ζ* (=0.27) and *χ* (=0.037) are correction factors determined by fitting experimental data, *μ* (~0.00025 [32]) is the friction coefficient, *E* is the whole-cell elasticity (0.300 kPa for MCF-7 [8]), and *ν* (~0.4 [33]) is the Poisson’s ratio of the cell. This model captures the interplay between channel geometry, cell size, and elasticity, enabling estimation of biophysical parameters from electrical signal features during transit.

## 4. Results and Discussion

### 4.1. System Design

**Figure 2:**
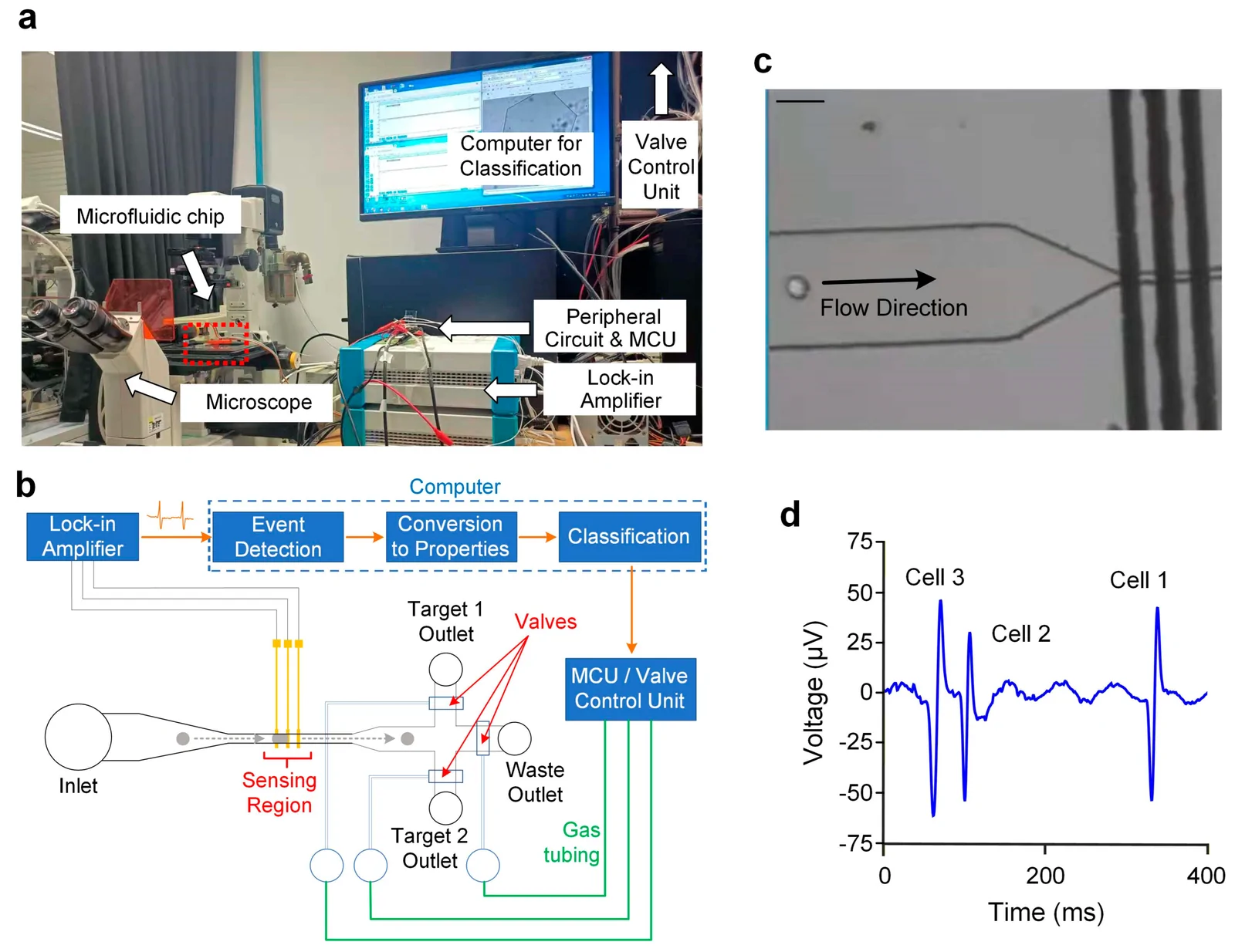
Illustration of system setup. (**a**) System settings. The microfluidic chip is connected to tubing for biosample access and a printed circuit board for the subsequent signal acquisition by a computer communicating with a lock-in amplifier. (**b**) Schematic diagram and workflow of the system platform. (**c**) Micrograph of a cell entering a constriction with sensing electrodes. Scale bar: 50 μm. (**d**) Measured voltage signal when a cell is passing through the channel.

The microfluidic biophysical cell phenotyping and sorting platform is illustrated in Figure 2a, with the implementation workflow shown in Figure 2b. The device incorporates a constricted microchannel designed to induce controlled deformation as cells transit. For human epithelial cells (10–26 μm in diameter), the channel height was set to 40 μm, and the constriction width was fixed at 8 μm (Figure 2c), ensuring cytoplasmic deformation while preserving nuclear integrity (typically <8 μm) [7,9].

Impedance-based electrokinetic sensing forms the core of the measurement scheme. Electrodes are positioned more than 100 μm downstream from the constriction entrance to allow flow stabilization prior to signal acquisition, ensuring reliable measurements. These electrodes are connected to a differential circuit and a lock-in amplifier. A reference signal at 500 kHz is applied to probe alpha dispersion effects [32,34] such that the measured impedance is sensitive to both cell size and deformation. As each cell passes through the sensing zone, signal features such as peak-to-peak voltage and peak period are recorded, serving as indicators of deformation extent. Data streams are transmitted in real time to a computer for processing (Figure 2d). The key experimental parameters of the microfluidic biophysical cell phenotyping and sorting platform are listed in Table 1. Furthermore, the constriction has a downstream length of 2 mm after the electrode site, offering sufficient window of >200 ms (the cell transit speed is typically <10 mm/s) for the required computation for measurement, cell classification, and sorting module actuation.

A custom software pipeline automatically processes signals and converts them into biophysical parameters for individual cells, enabling automated, label-free analysis. The architecture integrates two neural networks: one for event detection and another for property estimation. Classification results are then communicated to a downstream microcontroller unit (MCU) to actuate pneumatic microvalves for sorting. These valves operate by applying gas pressure to overhead channels [27], enabling precise gating of flow paths. By opening the designated outlet and closing others, identified cells are directed to their target collection channels.

### 4.2. Event and Feature Extraction Algorithms

**Figure 3:**
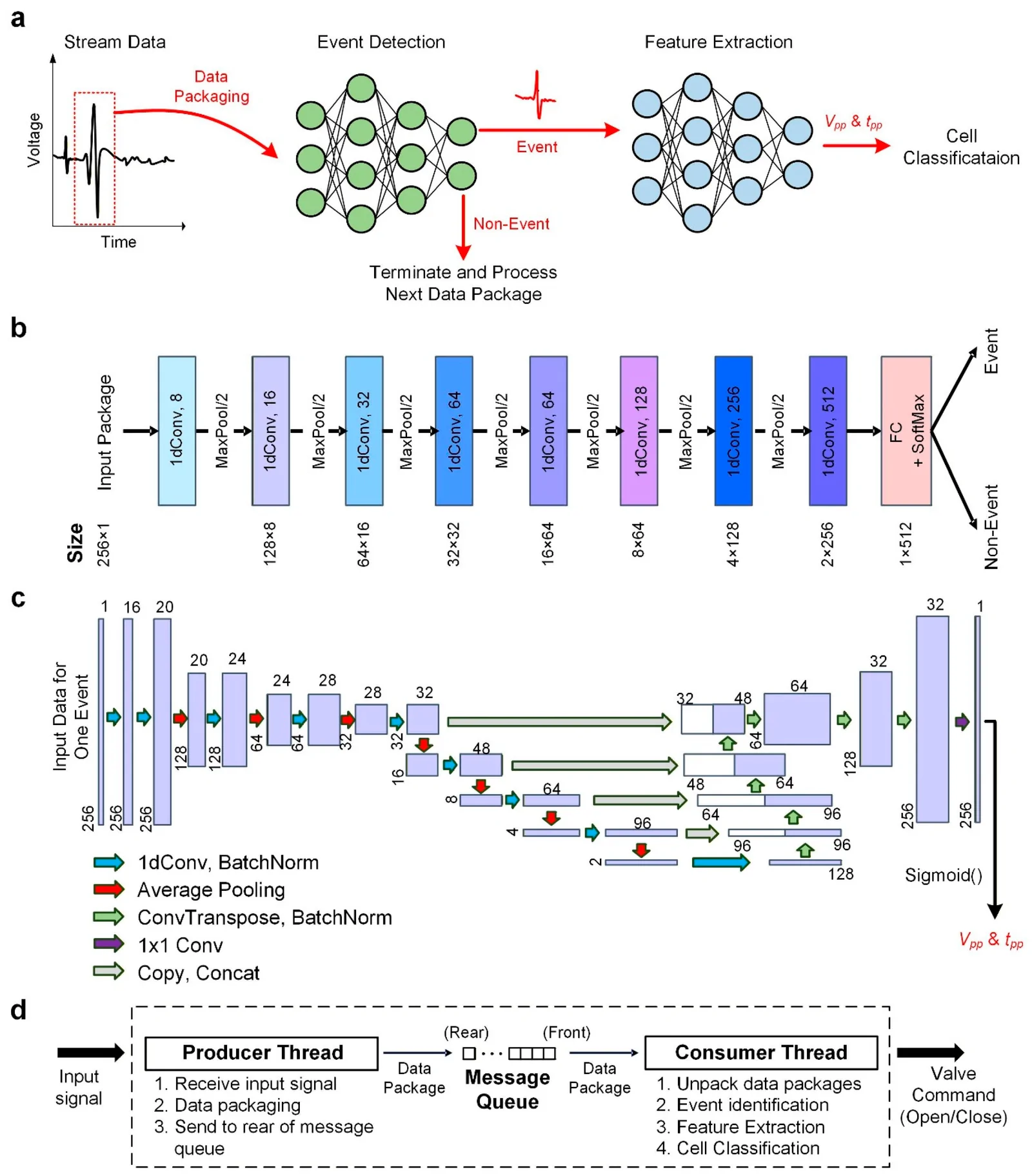
Cell event detection and feature extraction. (**a**) Overview of data analysis workflow. (**b**) Structure of the signal classification network. (**c**) Structure of the feature extraction network. (**d**) Architecture of the proposed parallel real-time data processing computational framework.

Neural networks were developed to perform event detection, enabling signal/noise classification and peak segmentation, as illustrated in Figure 3a. Electrical signals corresponding to cell passage are packaged and fed into a pre-trained model for recognition. The network identifies segments associated with individual cell events and extracts key features, including event duration and peak-to-peak voltage.

To classify whether a signal package represents a valid cell event, we implemented a VGG-inspired architecture [35] combining 1D convolutional neural networks (CNNs) with a fully connected network (FCN) (Figure 3b). The classification network comprises seven convolutional layers (kernel size = 7, padding = 3), followed by ReLU activation and max pooling to reduce feature dimensions. After convolution, a fully connected layer and SoftMax function perform binary classification into ‘event’ or ‘non-event’. To train the CNN prior to practical implementation, raw electrical recordings were manually reviewed and annotated to generate training labels. Signal packages containing valid cell transit events were labeled as positive events, whereas background fluctuations were labeled as non-events. For pulse segmentation, the starting and ending positions of each cell transit event were manually annotated to provide ground truth masks. The complete dataset was partitioned into training, validation, and testing subsets prior to model development. Network training was performed using a batch size of 6 and an initial learning rate of 1 × 10^−5^. The learning rate was reduced by a factor of 10 after every 100 epochs. Validation was performed after epoch 50, epoch 100, and every five epochs thereafter. The model achieving the best validation performance was selected for subsequent testing and real-time implementation.

For feature extraction, a modified U-Net architecture [36] with 1D CNN layers was adopted (Figure 3c). This network integrates down-sampling and up-sampling stages. Down-sampling employs convolutional layers (kernel size = 7, padding = 3) with batch normalization to stabilize training and improve convergence. Average pooling replaces max pooling to expand the receptive field. Up-sampling uses transpose convolution to restore feature vectors to their original length. Consistent with U-Net principles, skip connections concatenate shallow and deep features, enhancing peak detection accuracy. The final output assigns probabilities (0–1) to each signal point, indicating its likelihood of belonging to a peak region, enabling precise segmentation. To train the feature extraction network, individual sampling points in the training waveforms were manually annotated as either belonging to a cell transit pulse or background signal. Training utilizes binary segmentation masks generated from manually labeled pulse boundaries. The dataset was divided into training, validation, and testing subsets. Network optimization was performed using the Adam optimizer with an initial learning rate of 1 × 10^−5^ and a batch size of 6. Learning rate decay by a factor of 10 was applied after every 100 epochs. The model exhibiting the best validation performance was selected for deployment. The trained U-Net generated probability maps indicating pulse membership for individual sampling points, allowing robust determination of pulse boundaries and subsequent extraction of the peak-to-peak voltage (*V_pp_*) and peak period (*t_pp_*).

To support real-time operation, we implemented a parallel software architecture (Figure 3d) based on the producer–consumer pattern. This design decouples data acquisition from processing, preventing bottlenecks and maximizing throughput. The producer thread collects real-time signal streams from the lock-in amplifier via a Python API, packages them for neural network input, and places them in a message queue. The consumer thread retrieves these packages, processes them through the pre-trained models for feature extraction, and sends classification results to the microcontroller unit (MCU) for downstream sorting.

In the aspect of timing of the real-time sorting pipeline, the event detection neural network required approximately 2 ms per signal package, while feature extraction required approximately 4 ms. Computation of cell biophysical properties and threshold-based classification required less than 1 ms. Together with microcontroller communication, the total computational latency was approximately 7 ms. Considering an estimated pneumatic valve response time of 20–50 ms based on our previously reported analysis [37], the overall system latency remained below 60 ms. This latency is substantially shorter than the cell transit time (>200 ms as explained above) between the sensing region and the downstream sorting junction, thereby enabling successful real-time sorting. In the current system setting, the bottleneck of the latency time is the pneumatic valve response time, limiting the throughput of cell sorting to be ~10^2^ cells/min.

### 4.3. Cell Phenotyping and Classification

Recalling that lock-in amplification [26] with three electrodes per region was used (Figure 2b), electrode ‘2’ receives an oscillating voltage (|*V_s_*| = 1 mV at 300 kHz for alpha dispersion), while electrodes ‘1’ and ‘3’ connect to external resistors (100 Ω) at *V*_+_ and *V*_−_ nodes. Our recent study [29] reveals that the detected peak-to-peak voltage (*V_pp_*) is proportionally to *D_c_*, i.e., *V_pp_* ≈ *ξD_c_*, where *ξ* is determined by many factors including the measurement configurations, which are calibrated for this work. On the other hand, transit speed is approximated as *v_c_* ≈ *X_E_*/*t_pp_*, where *X_E_* is the center-to-center distance of two adjacent electrodes, and *t_pp_* is the time between up-peak and down-peak of a cell-passage-induced signal (Figure 2d). We can then apply the physical model for extracting *D_c_* and *E* from raw electrical signals. *D_c_* is converted from the measured *V_pp_*, whereas *v_c_* is computed by *X_E_*/*t_pp_*. *E* is then derived from *v_c_* and *D_c_* using the model described in the previous section (Equation (2)).

**Figure 4:**
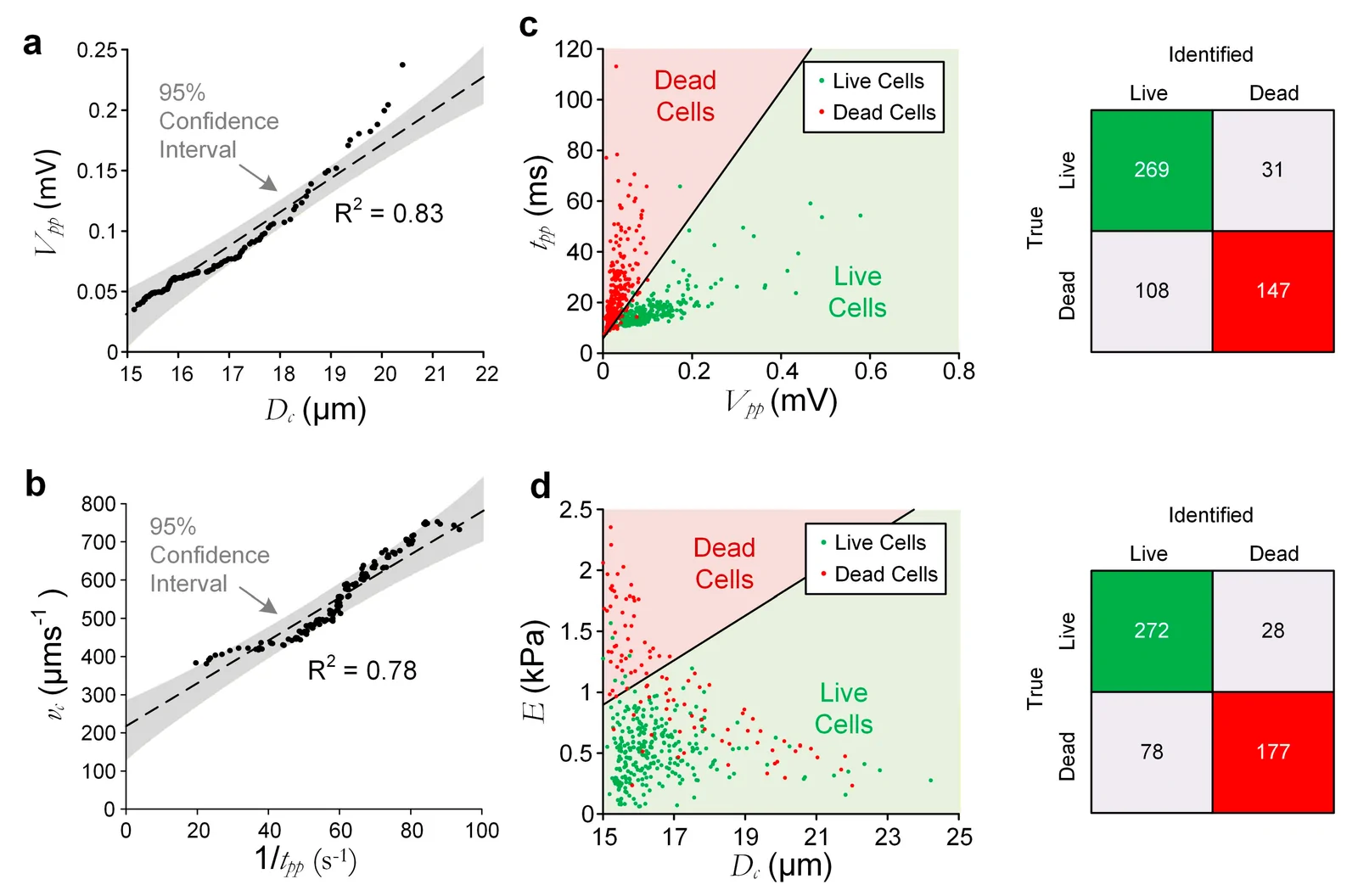
Results of calibration and experimental validation of measurement of cancer-related cell properties. (**a**) Calibration of *V_pp_* as proportional to *D_c_* along the constriction microchannel. *n* = 116 from four independent experiments. (**b**) Calibration of *v_c_* as proportional to 1/*t_pp_* along the constriction microchannel. N = 116 from four independent experiments. (**c**) Scatter plot of transit time and voltage measurement of dead (N = 265) and live cells (N = 290) (left), and confusion matrix (right). (**d**) Scatter plot of *E* vs. *D_c_* of dead (N = 265) and live cells (N = 290) (left) and confusion matrix (right).

Furthermore, we considered live (*n* = 290) and fixed/dead (*n* = 265) MCF-7 cells for their dominant cell properties. We injected the live cells and dead cells separated and hence the cell viability of each measured cell is certain. Each cell group was adapted to the real-time cell sensing. Overall, fixed cells have a slightly smaller cell size (14.90 ± SEM0.12 μm) than the live cells (16.47 ± SEM0.08 μm) but larger elastic modulus (1.898 ± SEM0.067 kPa) than the live cells (527 ± SEM0.015 kPa), agreeing with the previously reported trends [38]. The *p*-values for these two comparisons are both <0.0001, obtained using Student’s unpaired two-tailed *t*-test. The relationships between *V_pp_* and *D_c_* for the live and dead cells are determined through prior calibration (Figure 4a) by comparing the measured *V_pp_* and the corresponding values of *D_c_* directly observed from micrographs. *v_c_* observed from time-lapsed micrographs also correlates with 1/*t_pp_* (Figure 4b). Next, we implement biosensing of individual cells in the biosample and the signal features. A two-dimensional scatter plot of transit time versus voltage is shown in Figure 4c. *V_pp_* for dead cells is significantly lower than that for live cells, primarily owing to the distinct electrical and mechanical properties caused by the cell fixation process [38]. It is also observed that the dead cells exhibit longer transit times across the electrode region (23.83 ± SD10 ms) compared to live cells (16.80 ± SD9 ms), possibly attributed to the less deformable cytoskeleton of the dead cells.

We have implemented the threshold-based linear classifier for cell classification. This method involves searching for gradient and *y*-intercept of a boundary line for the maximum classification accuracy. The linear boundary parameters were determined using the measurement results as the dataset and subsequently evaluated using the same dataset. No additional ‘testing’ data were used during parameter optimization. Although a boundary between live and dead cells can be observed in Figure 4c, it is not yet practical to differentiate physical properties between the two cell groups given the classification accuracy, defined as the number of corrected identified cells divided by the total cell number, is 75%. Alternatively, the cell signals were converted to *D_c_* and *E* for downstream comparison and classification, by constructing another threshold-based linear classifier. Results (Figure 4d) demonstrate that the converted cell properties can still reveal different regions of the live and dead cells. Briefly, the dead cells are smaller in size and larger in whole-cell elasticity than the live cells. We further conduct cell classification of the two cell types (live cells and dead cells) with accuracy of 81%. This improvement indicates that the converted distribution of *D_c_* and *E* would facilitate a linear classification cutoff condition. It should be mentioned that the cell classification accuracy is constrained by the cell property distribution. For instance, our recent study on classification of non-cancerous/cancer breast cell lines [29] can obtain and accuracy of >95.45%, similar to the values reported by other research group working on cancer cell classification [16,17].

### 4.4. Cell Sorting

**Figure 5:**
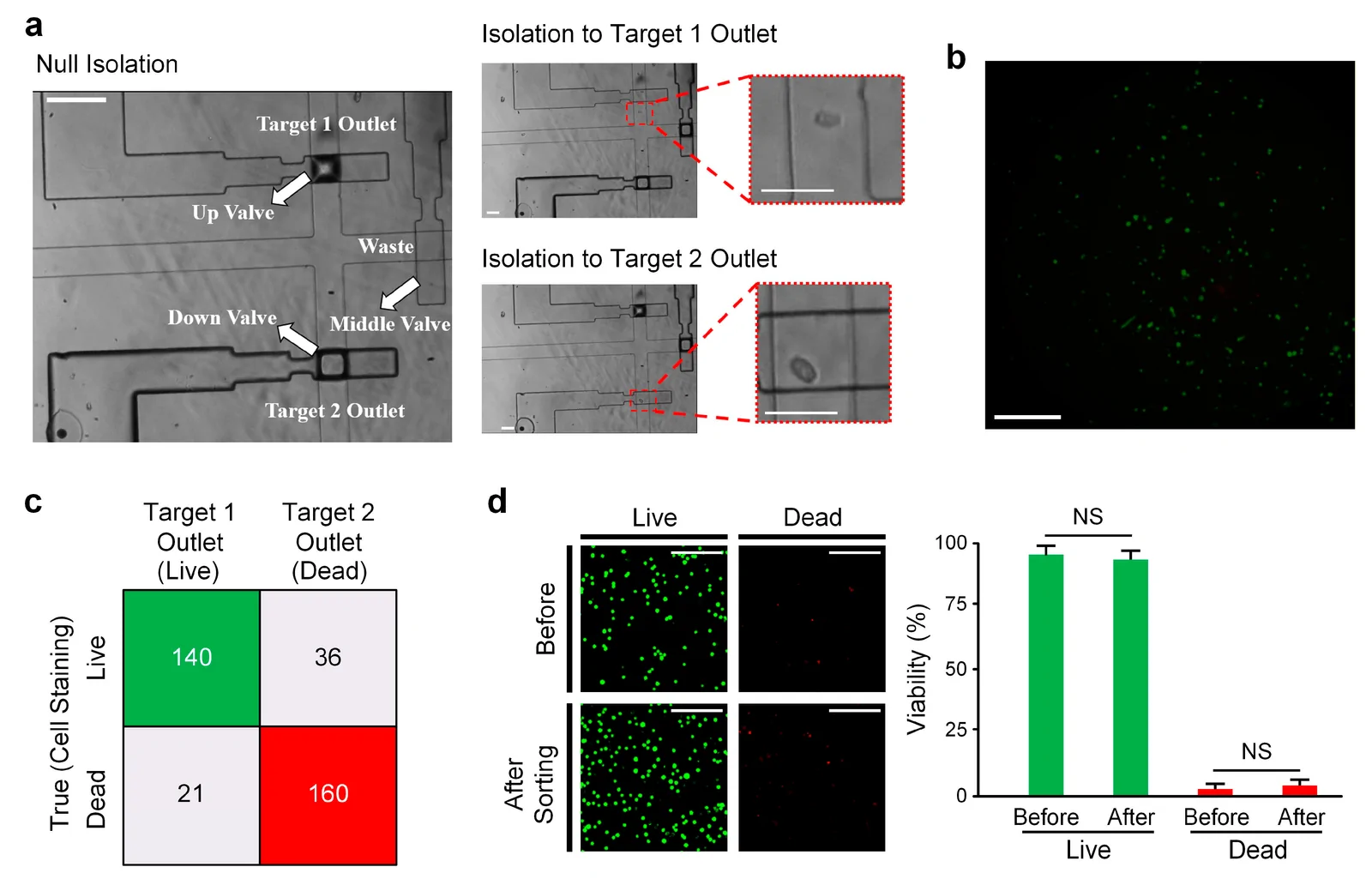
Results of the cell sorting experiment. (**a**) Illustration of the cell sorting process using the microvalves with three modes: (1) Null isolation with flows to waste outlet, (2) isolation to Target 1 outlet, and (3) isolation to Target 2 outlet. (**b**) Fluorescence micrograph of the stained MCF-7 cells collected for Target 1 outlet for sorted live cells. Green for live cells and red for dead cells. Scale bar: 500 μm. (**c**) Live and dead cell counts from stained cells collected at outlets of the microfluidic sorting device. (**d**) Cell viability before (*n* = 60) and after (*n* = 60) measurement and sorting using the microfluidic platform. Captured fluorescence micrographs (**left**) and the quantified results (**right**) are provided. Three repeated experiments (*n* = 20 each) were performed. There is no statistically significant difference between before and after measurements and sorting. Scale bar: 250 μm. Statistical insignificance is determined by a *p*-value ≥ 0.05 obtained using Student’s unpaired two-tailed *t*-test.

Real-time sensing and sorting were implemented using a mixed population of live and dead MCF-7 cells (~50% each) to create samples with distinct elasticity ranges. Following classification based on extracted biophysical properties (*D_c_* and *E*) using the same boundary as presented in Figure 4d, decision commands were transmitted to the MCU to control pneumatic microvalves for channel gating (Figure 5a). Three operational modes were defined: (1) null isolation, (2) isolation A, and (3) isolation B. When a target outlet was selected, its corresponding microvalve opened while the remaining valves closed, directing the identified cell to the designated collection channel.

To validate the cell sorting performance, we further performed the cell sorting experiments with pre-stained MCF-7 cell mixture using the live/dead cell imaging kit (R37601, Invitrogen). It was noted that classification accuracy reflects the performance of the software-based decision algorithm, whereas sorting accuracy reflects the end-to-end system performance including signal acquisition, classification, communication with the microcontroller, pneumatic valve actuation, and outlet collection. The sorted cells from either Target 1 outlet or Target 2 outlet were then pipetted onto a glass slide, followed by placing a coverslip overhead. The fluorescent micrographs were captured, and the live/dead MCF-7 cells were counted accordingly. An example of sorted cells at Target 1 outlet is shown in Figure 5b. The results show that the achieved sorting accuracy was 84% (Figure 5c), defined as the ratio of correctly sorted cells (140 live cells and 160 dead cells) and the total number (357) of cells. This value is close to the classification accuracy (81%) reported in Figure 4c. This agreement suggests that the minor flow fluctuation caused by microvalve movement in the cell sorting operation does not affect the cell classification performance. This also confirms the ability of the system to make sorting decisions before cells reach the branching region. Based on this performance, most cells can be reliably directed to their target outlet using the pneumatic valve module. Additionally, we examined any reduction in cell viability caused by the microfluidic process. We applied live cells and compared one cell group without the microfluidic processes and another group after the microfluidic measurement and sorting process. We have confirmed the viability of the measured cells for further analyses and cell processes was presented in Figure 5d.

## 5. Conclusions

We have developed an automated microfluidic platform that integrates impedance-based sensing with constriction microchannel architecture for real-time, label-free cytometry and sorting of single cells. The system enables accurate estimation of cell size and whole-cell elasticity through electrical signal analysis, supported by a biomechanical model and validated against imaging measurements. Experimental results demonstrate reliable phenotyping and sorting of live and dead MCF-7 cells, achieving sorting accuracy of 84%. This approach offers a cost-effective and scalable solution for biophysical analysis for individual floating cells. Further implementation of sorting different cell types with different populations would characterize the sorting performance in more detail. Future work will focus on extending the platform to heterogeneous cell populations, improving analytical throughput, and enhancing sorting precision through advanced signal processing and microfluidic control.

## Figures and Tables

**Table 1:** Summary of key operating parameters of the microfluidic platform.

Parameter	Value	Parameter	Value
Channel height	40 μm	Excitation frequency	500 kHz
Constriction width	8 μm	Excitation voltage	1 m (*V_pp_*)
Electrode width	8 μm	Sampling rate	~50 kHz
Electrode gap	8 μm	Valve operating pressure	20 kPa
Electrode center-to-center spacing	16 μm	Cell concentration	10^4^ cells/mL

## Data Availability

All data needed to evaluate the conclusions in the paper are present in the paper. Specific data will be made available on request.
